# Emodin Augments Cisplatin Cytotoxicity in Platinum-Resistant Ovarian Cancer Cells via ROS-Dependent MRP1 Downregulation

**DOI:** 10.1155/2014/107671

**Published:** 2014-12-14

**Authors:** Jun Ma, Jie Yang, Chao Wang, Nan Zhang, Ying Dong, Chengjie Wang, Yu Wang, Xinjian Lin

**Affiliations:** ^1^Department of Obstetrics and Gynecology, Renji Hospital, School of Medicine, Shanghai Jiaotong University, Shanghai 200127, China; ^2^Department of Cell Biology, Key Laboratory of the Education Ministry for Cell Differentiation and Apoptosis, Institute of Medical Sciences, School of Medicine, Shanghai Jiaotong University, Shanghai 200025, China; ^3^Department of Medicine and UC San Diego Moores Cancer Center, University of California, La Jolla, San Diego, CA 92093, USA

## Abstract

The intracellular level of reactive oxygen species (ROS) is closely associated with chemosensitivity of cancer cells. Overexpression of ATP binding cassette transporter MRP1 is correlated with resistance to platinum drugs. In this study, we tested the hypothesis that emodin, a potent ROS generator, may increase sensitivity of cisplatin-(cDDP-) resistant ovarian carcinoma cells to cDDP cytotoxicity via ROS-mediated suppression of MRP1 expression. Using the isogenic pair of the human ovarian carcinoma cell line COC1 and its cDDP resistant variant COC1/DDP, we found that ROS level in the cDDP-sensitive ovarian cancer cells was significantly higher than that in the cDDP-resistant cells. Emodin enhanced ROS production in COC1/DDP cells and consequently sensitized them to cDDP-induced apoptosis. These effects were reversed by addition of the antioxidant N-acetyl-L-cysteine (NAC). Cotreatment with emodin and cDDP inhibited the tumor growth *in vivo* by increasing tumor cell apoptosis. The emodin-enhanced cDDP cytotoxicity was attributable to downregulation of multidrug resistance-related protein 1 (MRP1) expression. Together, these results suggest that emodin could act as an adjunct to enhance the anticancer effect of cDDP likely through ROS-related downregulation of MRP1 expression, and may be of therapeutic potential in cDDP-refractory ovarian carcinomas.

## 1. Introduction

Ovarian cancer (OC), consisting predominantly of carcinomas, is the most lethal gynecologic malignancy and currently ranks fifth in causing cancer-related deaths among women [1]. The poor survival of OC patients is attributed to diagnosis at advanced stage and resistance to chemotherapy [2]. Cisplatin (cDDP) is an effective first-line therapy against ovarian carcinoma both in adjuvant treatment and in the care of patients with advanced disease [3, 4]. However, its clinical efficacy is limited by the rapid development of resistance. Most tumors that are initially sensitive to this drug become resistant over the course of four to six cycles of treatment and when this occurs, subsequent therapy with other agents is generally of limited value and the patient eventually dies [5]. Therefore, there is an urgent need to develop novel approaches and treatment options to overcome the acquired drug resistance.

cDDP is widely believed to kill cells predominantly by forming adducts in DNA that block transcription and DNA replication. Extensive studies on cDDP resistance have revealed that it is multifactorial in nature with no single overarching mechanism predominating even within the same histological type of tumor. Novel insights into molecular mechanisms of resistance are important to the goal of identifying patients whose tumors have a high probability of responding to cDDP and avoiding administration of this drug to patients unlikely to benefit from treatment. Mechanisms implicated in cellular resistance include reduced drug uptake, increased drug efflux, increased DNA repair, increased tolerance of DNA damage, and aberrations in apoptosis pathways [6]. Cancer cells retain the important mechanism of self-protection through the activity of multiple drug exports. The multidrug resistance phenotype is frequently associated with overexpression of membrane pumps that efflux anticancer drugs from the cytoplasm. Studies have implicated that impaired accumulation of cDDP in the cDDP-resistant cells is associated with increased expression of members of the ATP binding cassette (ABC) family of transporters such as multidrug resistance-associated protein 1 (MRP1) [7–10], a member of glutathione (GSH) conjugate export pump (GS-X pump).

The intracellular level of reactive oxygen species (ROS) has been found to be closely related to the chemosensitivity of cancer cells. Increase in ROS production is known to enhance cytotoxic effects of various anticancer drugs whereas cells with a lower ROS level appear less responsive to chemotherapy [11]. In this regard, manipulation of oxidation-reduction (redox) status of cancer cells to sensitize them to chemotherapeutic drugs is being exploited as a potential therapeutic and resistance-circumventing strategy. Emodin (1,3,8-trihydroxy-6-methyl anthraquinone), a natural anthraquinone derivative, is an ROS generator [12] and has been shown to enhance the cytotoxicity of arsenic trioxide selectively in human cervical cancer HeLa cells and human leukemia U937 cells via increased generation of ROS and ROS-mediated inhibition of survival signaling [13]. More recently, Wang et al. showed that emodin can effectively sensitize human gallbladder cancer SGC996 cells, which are intrinsically resistant to many cancer drugs, to platinum drugs through GSH depletion and MRP1 downregulation [14]. Despite the fact that emodin has been applied as a sensitizer for cytotoxic therapies in multiple cell models, to the best of our knowledge, no study has explored its possible sensitizing activity and underlying mechanisms in chemoresistant ovarian cancer cells. Our present study was based on the hypothesis that emodin can sensitize platinum-resistant ovarian cancer cells to cDDP-induced apoptosis through elevation of intracellular ROS and downregulation of the GSH conjugate exporter MRP1.

## 2. Materials and Methods

### 2.1. Cell Culture and Reagents

The parental human ovarian adenocarcinoma cell line COC1 and its cDDP-resistant derivative COC1/DDP were purchased from CCTCC (China Center for Type Culture Collection). COC1/DDP subline was developed by continuous stepwise selection in increasing concentrations of DDP with 6.5-fold more resistance to cDDP than the parent cell line COC1 as measured by the ratio of IC_50_ values [15]. COC1 and COC1/DDP cells were maintained in RPMI-1640 medium (GibcoBRL, Gaitherburg, MD) supplemented with 10% fetal bovine serum, 100 U/mL penicillin and 100 *μ*g/mL streptomycin under a humidified atmosphere with 5% CO_2_ at 37°C. Cisplatin (cDDP) were purchased from Qilu Pharmaceutical Co, Ltd (Ji Nan, China). Emodin and N-acetyl-L-cysteine (NAC) were obtained from Sigma-Aldrich (St. Louis, MO, USA).

### 2.2. Cell Apoptosis Analysis

Cells were treated with drugs for 24 h and the fraction of apoptotic cells was assessed with flow cytometry using annexin V-fluorescein isothiocyanate (Annexin V-FITC)/propidium iodide (PI) kit (BD Pharmingen, San Diego, CA). Samples were prepared according to the manufacturer's instructions and analyzed by flow cytometry on FACS Calibur.

### 2.3. ROS Measurement

2,7-Dichlorodihydrofluorescein diacetate (DCFH-DA, Sigma) was used as ROS capture in the cells. The average fluorescent intensity of 2,7-dichlorofluorescein (DCF) is proportional to intracellular ROS levels. Cultured cells were exposed to drugs and 10 *μ*M of DCFH-DA at 37°C for 15 min. After they were washed once with ice-cold PBS, cells were harvested and kept on ice until undergoing flow cytometric analysis.

### 2.4. Reverse Transcription PCR (RT-PCR)

Total RNA was isolated from cells by TRIzol Reagent (Invitrogen, Carlsbad, CA, USA). First-strand cDNA was synthesized using random primers and AMV reverse transcriptase (Promega, Madison, WI, USA). The forward and reverse primers for MRP1 and glyceraldehyde-3-phosphate dehydrogenase (GAPDH) were, respectively, 5′-TGGTGGGCCTCTCAGTGTCTTA-3′ and 5′-TCGGTAGCGCAGGCAGTAGTTC-3′ and 5′-TGGGGAAGGTGAAGGTCGG-3′ and 5′-CTGGAAGATGGTGATGGGA-3′. The thermal cycling conditions were 94°C 30 sec, 58°C 30 sec, and 72°C 30 sec for 34 cycles. The PCR products were run on 1.5% agarose gel and the density of the bands on the gel was quantified by densitometry using Tocan gel imaging analysis system. Gene expression was presented as the relative yield of PCR product from the MRP1 gene to the reference GAPDH gene. Samples were prepared in triplicate with 3 independent sample sets being analyzed.

### 2.5. *In Vivo* Efficacy Study

All work performed with animals was in accordance with and approved by the Institutional Animal Care and Use Committee at the Shanghai Jiao Tong University School of Medicine. Six to 7 week old female BLAB/c-nu/nu mice (Shanghai Experimental Animal Center, Shanghai, China) were subcutaneously inoculated with 1 × 10^7^ COC1/DDP cells bilaterally into the left and right flank region. Three days after inoculation, the tumor-bearing mice were randomly divided into 4 groups (8 mice per group) and treated with saline, emodin (50 mg/kg), cDDP (1 mg/kg), or emodin (50 mg/kg) plus cDDP (1 mg/kg) every two days for 18 days by the i.p. route. Tumor volume was determined every other day by the formula: volume = (length × width^2^)/2 and plotted as a function of time to generate the* in vivo* growth curves. The mice were sacrificed 21 days after tumor implantation and the tumor tissues were sectioned for TUNEL staining according to the manufacturer's protocol (*In Situ* Cell Death Detection Kit; Roche Diagnostics, Indianapolis, IN, USA).

### 2.6. Statistical Analysis

Data are presented as mean ± SD. SPSS 17.0 software was used for statistical analysis. ANOVA (analysis of variance) was applied for comparison of the means of two or multiple groups, in which SNK (Student-Newman-Kewls) was further used for comparison of each two group. A value of *P* < 0.05 was considered significant.

## 3. Results

### 3.1. cDDP-Resistant Cells Display Lower Levels of ROS

**Figure 1:**
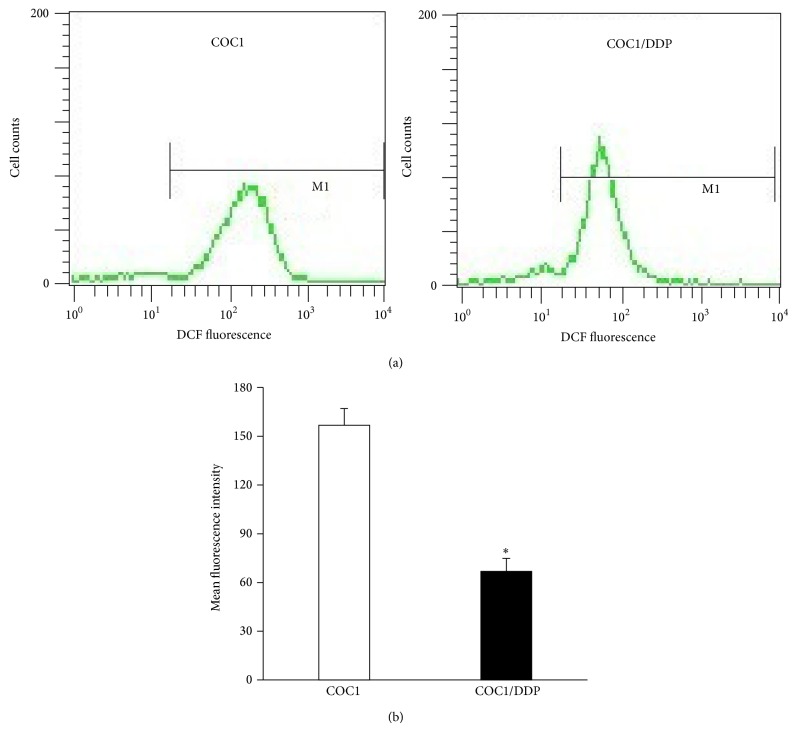
Intracellular ROS levels in cDDP-sensitive (COC1) and cDDP-resistant (COC1/DDP) ovarian cancer cells. (a) A flow cytometric analysis of DCFDA staining in the cells with an analytical gate (M1) set at the same position. (b) The data from three independent experiments are expressed as the mean fluorescence intensity ± SD. ^*^ *P* < 0.05.

The regulation of oxidative stress is an important factor in both tumor development and response to anticancer therapies. To determine whether cDDP resistance was a result of altered intracellular ROS levels, ROS was measured in COC1 and COC1/DDP cells. In three independent experiments, each performed with triplicate samples, ROS levels were determined using the fluorescent probe DCFDA and the fluorescence of cells was quantified by flow cytometry. The mean fluorescence intensity was 156.7 ± 10.3 in COC1 cells and 66.8 ± 8.0 (SD) in COC1/DDP cells (Figures 1(a) and 1(b)), representing a 2.3-fold (*P* < 0.05) reduction in ROS levels when COC1 cells were selected for resistance to cDDP. Thus, at least part of the acquired cDDP resistance can be accounted for by a decrease in the intracellular ROS accumulation.

### 3.2. Emodin Enhances cDDP-Induced Apoptosis in cDDP-Resistant Ovarian Cancer Cells

**Figure 2:**
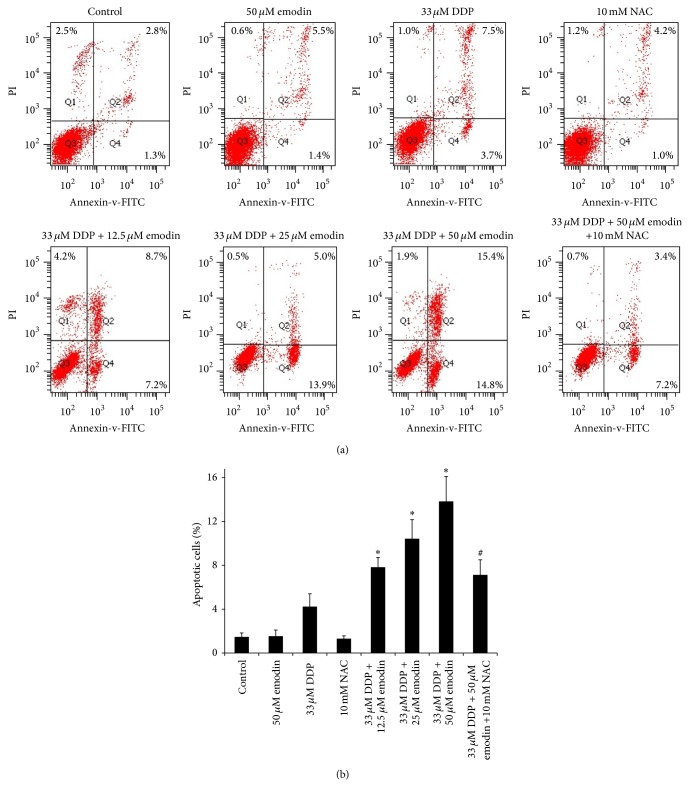
Effect of emodin on cDDP-induced apoptosis in cDDP-resistant COC1/DDP cells. (a) Representative density plots of flow cytometric analysis on the fraction of apoptotic cells 24 h after the indicated treatments detected with annexin V/propidium iodide. (b) The histogram represents the mean values of three independent experiments. ^*^ *P* < 0.05 versus cDDP-only treated cells; ^#^ *P* < 0.05 versus the 30 *μ*M DDP + 50 *μ*M emodin cotreated cells.

Given the observation that resistance to cDDP was linked to lower intracellular ROS levels and that high levels of ROS are generally detrimental to cells, we next sought to determine whether addition of emodin, a well-known ROS generator, could sensitize the cDDP-resistant COC1/DDP cells to the cytotoxic effects of cDDP. To this end, annexin-V/PI dual staining for flow cytometric analysis was used to measure an early event of apoptosis. As shown in Figure 2, emodin alone caused no appreciable apoptosis as compared to the untreated control cells. However, it significantly potentiated cDDP-induced apoptosis of the COC1/DDP cells in a dose-dependent manner (*P* < 0.05). Intriguingly, the enhancing effect of emodin at 50 *μ*M on cDDP-evoked apoptosis was largely suppressed by an antioxidant, NAC, as concurrent treatment with NAC reduced the frequency of apoptotic cells by 48.6 ± 7.5% (*P* < 0.05 versus the 33 *μ*M DDP + 50 *μ*M emodin combination) to a level of 1.7 ± 0.6-fold above that in the cDDP alone treated cells (7.1 ± 1.4% in the three-drug combination versus 4.2 ± 1.2% in the cDDP alone, *P* > 0.05).

### 3.3. Sensitization of cDDP-Resistant Cells to cDDP by Emodin Is Associated with ROS Generation

**Figure 3:**
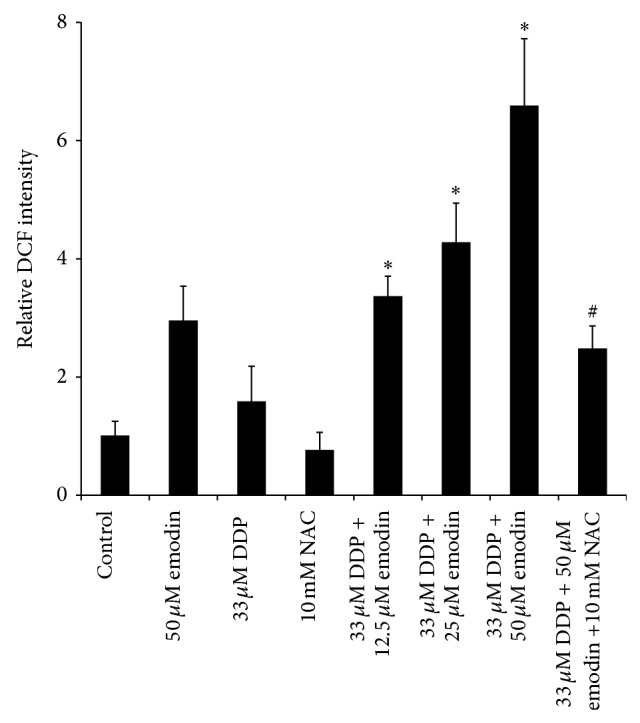
Production of ROS in response to cDDP and emodin. COC1/DDP cells were treated with 50 *μ*M emodin, 33 *μ*M cDDP, 10 mM NAC, 12.5, 25, or 50 *μ*M emodin + 33 *μ*M cDDP, or 50 *μ*M emodin + 33 *μ*M cDDP + 10 mM NAC for 24 h and then incubated with DCFDA for 15 min. Cellular ROS level was reflected by DCF intensity and expressed as a fold change of the DCF intensity relative to that in the untreated control cells. Values represent mean ± S.D. of three separate experiments performed in duplicate. ^*^ *P* < 0.05 versus cDDP-only treated cells; ^#^ *P* < 0.05 versus the 30 *μ*M DDP + 50 *μ*M emodin cotreated cells.

Generation of ROS is known to be an early signal that mediates apoptosis [16]. Acute apoptosis by cDDP has been shown to be more closely associated with cytoplasmic generation of ROS than DNA damage [17]. To verify that emodin-enhanced cDDP cytotoxicity is mediated through overproduction of ROS, intracellular ROS levels, as reflected by DCF fluorescence intensity, were determined following the treatment of COC1/DDP cells with emodin, cDDP, NAC, emodin + DDP, or emodin + cDDP + NAC for 24 h.  Figure 3 shows that the relative DCF intensity largely mirrored the cDDP cytotoxicity pattern. Compared to the nontreated control, exposure of COC1/DDP cells to emodin resulted in a significant elevation of cellular ROS level (2.9 ± 0.6-fold, *P* < 0.05) while cDDP treatment induced less pronounced increase (1.6 ± 0.6-fold, *P* > 0.05). Remarkably, cotreatment with increasing concentrations of emodin and 33 *μ*M cDDP caused an emodin dose-dependent increase in ROS production (*P* < 0.05 versus cDDP alone). Similar to the cytotoxicity data, NAC significantly attenuated the two-drug (33 *μ*M DDP + 50 *μ*M emodin) potentiated ROS elevation by a factor of 2.6-fold (*P* < 0.05). These results indicate that enhancement of cDDP cytotoxicity by emodin is primarily dependent on ROS.

### 3.4. Emodin Increases the Therapeutic Efficacy of cDDP against cDDP-Resistant Tumors

**Figure 4:**
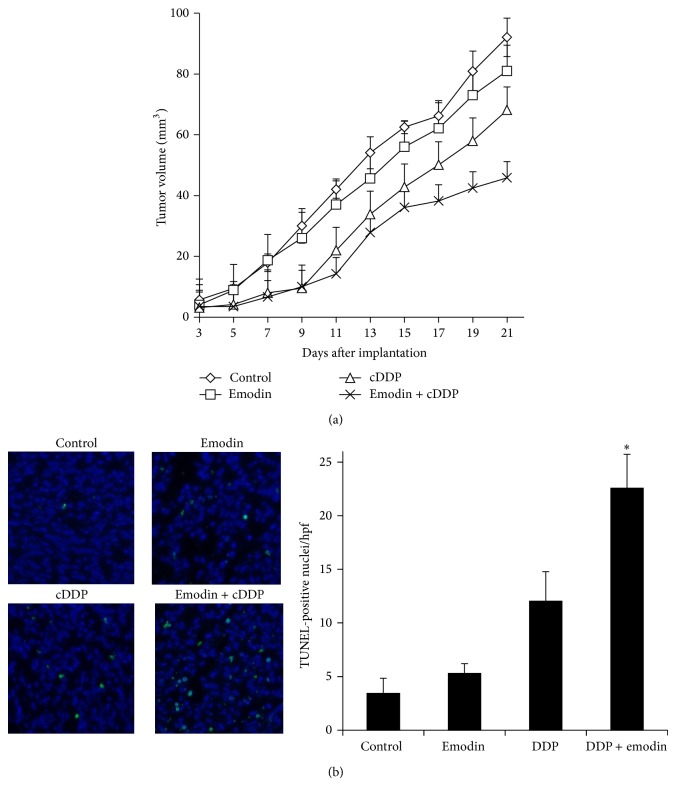
Effect of emodin on tumor responsiveness to cDDP* in vivo*. (a) cDDP-resistant COC1/DDP tumor growth curves after the mice were i.p. injected with the indicated drug(s) every 2 days for 18 days. Treatment was started on day 3 after tumor implantation. *N* = 8. (b) Numerical quantification of apoptosis in the COC1/DDP tumors by TUNEL staining. The average number of TUNEL-positive nuclei (stained green) per high-power field (hpf) was determined from eight tumors in each treatment group. ^*^ *P* < 0.05 versus cDDP-only treated group.

To determine whether the emodin-enhanced cDDP sensitivity measured* in vitro* translated into tumor responsiveness* in vivo*, the cDDP-resistant COC1/DDP cells were xenografted subcutaneously into BALB/c nu/nu mice which were then treated with either emodin or cDDP alone or the combination of the two drugs by i.p. injection every other day for 18 days.  Figure 4(a) shows that emodin had no antitumor activity while cDDP produced a moderate response in the COC1/DDP tumors. However, the combined therapy at the equitoxic doses of emodin and cDDP significantly increased the tumor responsiveness as compared to cDDP treatment alone by an analysis of the slope of the overall growth curves in repeated experiments (slope 2.7 ± 0.4 for the cotreatment versus 3.9 ± 0.6 for cDDP alone, *P* < 0.05). To examine the basis for the difference in tumor responsiveness to the treatment(s), COC1/DDP tumors were analyzed by the* in situ* TUNEL staining. As shown in Figure 4(b), emodin alone- and cDDP alone-treated tumors had an average of 5.3 ± 0.9 and 12.0 ± 2.8 TUNEL-positive nuclei per high-power field (hpf), respectively. In contrast, the two-drug cotreated tumors had an average of 22.6 ± 3.2 TUNEL-positive nuclei/hpf, representing a 1.9 ± 0.3-fold (*P* < 0.05) higher frequency of apoptotic cells in the cotreated tumor tissues than those treated with cDDP alone. Thus, consistent with its effect on cDDP cytotoxicity* in vitro*, emodin also enhanced the therapeutic efficacy of cDDP* in vivo* against the COC1/DDP tumor.

### 3.5. Emodin and DDP Cotreatment Downregulates MRP1 Expression

**Figure 5:**
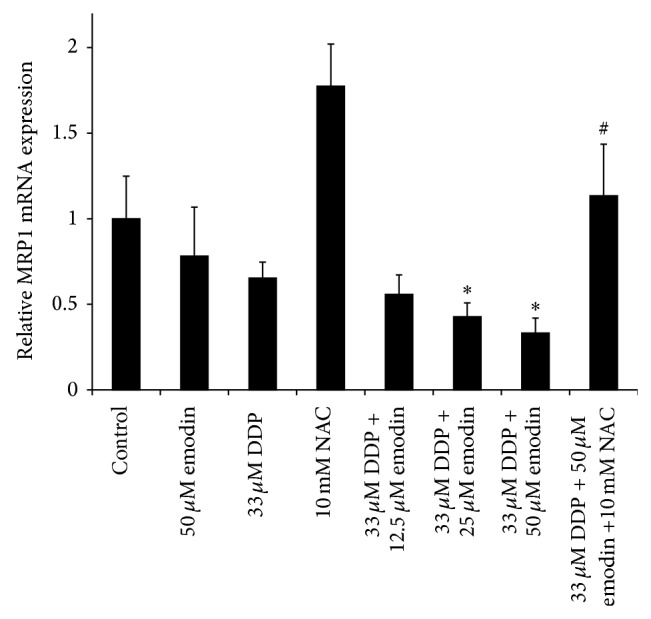
Expression of MRP1 mRNA in COC1/DDP cells exposed to cDDP or emodin. RT-PCR analysis was performed to assess MRP1 mRNA levels expressed as the fold change relative to that in the untreated control cells after normalization to GAPDH. Values are mean ± S.D. of three separate experiments performed in duplicate. ^*^ *P* < 0.05 versus cDDP-only treated cells; ^#^ *P* < 0.05 versus the 30 *μ*M DDP + 50 *μ*M emodin cotreated cells.

Based on previous reports indicating that some of the ABC transporters may be responsible for the defect in Pt-drug accumulation in resistant cells [8–10] as well as a recent study documenting that cotreatment with emodin could remarkably enhance chemosensitivity of platinum-resistant gallbladder cancer cells to platinum drugs via ROS-related mechanisms and downregulation of MRP1 [14], we examined whether the emodin-enhanced cytotoxic effect of cDDP in COC1/DDP cells was correlated with expression of multidrug resistance-related protein 1 (MRP1). As shown in Figure 5, under the same treatment schedules as used in the* in vitro* cytotoxicity assay, DDP alone slightly downregulated MRP1 expression whereas the expression of MRP1 was further reduced by cotreatment with emodin in a dose-dependent manner. To further document that redox state could account for the change in MRP1 expression, COC1/DDP cells were concurrently treated with 50 *μ*M emodin, 33 *μ*M cDDP, and 10 mM NAC. Cotreatment of cells with NAC attenuated suppression of MRP1 caused by cDDP in combination with emodin. These results suggest that enhanced cDDP cytotoxicity by emodin may be attributed to downregulation of the GS-X pump thus increasing cDDP intracellular concentration.

## 4. Discussion

Platinum-based chemotherapy is the most commonly used therapeutic approach for ovarian carcinomas in addition to surgical tumor debulking. Intracellular levels of ROS affect the cytotoxicity of a number of chemotherapeutic drugs including platinum-containing drugs [11, 18]. A ROS producer emodin has been shown to potentiate the cytotoxic effects of platinum drugs on prostate and gallbladder cancer cells involving ROS-related mechanisms and downregulation of multidrug resistance transporters [14, 19]. Here we show that combined treatment of emodin and cDDP induced a higher level of oxidative stress which was related to a more potent killing of cDDP-resistant ovarian cancer cells both* in vitro* and* in vivo* and stronger suppression of MRP1 expression.

Emodin, a naturally occurring anthraquinone, present in the roots and barks of numerous plants, is an active ingredient of various Chinese herbs including* Rheum officinale* and Polygonum cuspidatum medicine [14]. Its molecular structure is similar to that of 2,3-dimethoxy-1,4-naphthoquinone (DMNQ) [20], an agent that generates ROS intracellularly because its property of quinone and derived semiquinone, like mitochondrial ubiquinone, allows it to transfer electrons [21]. Oxidative stress is the cellular status resulting from overproduction of intracellular ROS and/or impaired function of the cellular antioxidant defense system [22]. It has been well established that the intracellular redox status plays a crucial role in cell survival and death and excessive ROS generation triggers downstream cellular and molecular events such as alterations of mitochondrial function and signal transduction leading to apoptotic cell death [19]. Pharmacological studies have demonstrated that emodin possesses antibacterial [15], anti-inflammatory [16], immunosuppressive [17], and anticancer effects [18]. While a link between its anticancer effect and ROS generation has been established, it should be noted that emodin alone requires a high dose to achieve its growth inhibitory effect or induction of cell death [13]. In the present study we found that 50 *μ*M emodin did not cause an appreciable cell death whereas the same concentration moderately increased intracellular ROS level. Many cancer therapeutic drugs can induce apoptosis by imposing oxidative stress and disrupting the intracellular redox balance. Treatment of the resistant COC1/DDP cells with 33 *μ*M cDDP also had little effect on cellular ROS level. Remarkably, combination treatment with both drugs elicited a marked elevation of the intracellular ROS level. Likewise, exposure to emodin plus cDDP significantly sensitized the original cDDP resistant COC1/DDP cells to the cytotoxic effect of cDDP. The emodin-enhanced cDDP cytotoxicity was apparently dependent on ROS generation since the augmentation of both ROS level and apoptosis by cotreatment with the two drugs was attenuated by the antioxidant NAC. Since the antioxidant thiol NAC acts by restoring the levels of intracellular GSH whose depletion is known to occur before the onset of apoptosis induced by various chemotherapeutic agents [23], it is conceivable that emodin potentiates cDDP-induced apoptosis in the cDDP-resistant ovarian cancer cells by depleting intracellular thiols. It is noteworthy that combination of cDDP with emodin led to the sensitization of COC1/DDP xenografts to cDDP treatment as evidenced by slower tumor growth and more apoptosis within the tumors. These results may provide a basis for the pharmacologic effect of emodin as a valuable cDDP therapeutic adjuvant for treatment of platinum-resistant ovarian carcinomas.

The therapeutic effect of cDDP is attributed to covalent adduct formation in DNA. Such DNA damage activates signals that trigger apoptosis in various solid tumor cells. However, only a small fraction of the intracellular cDDP can reach and bind to genomic DNA. A majority of fraction, approximately 60% of the intracellular cDDP, is conjugated with GSH to form GS-platinum complexes which mask cDDP cytotoxicity and eventually are transported out of cancer cells via the glutathione conjugate export pump [24]. As a type of GS-X pump, MRP1 is shown responsible for exporting cellular glutathione conjugation [14]. An association between increased expression of MRP1 and resistance to cDDP has been previously reported in a panel of lung cancer cell lines not selected* in vitro* for drug resistance [25]. Increased levels of MRP1 were also observed in ovarian carcinoma cells selectively resistant to oxaliplatin [10]. Thus, downregulation of MRP1 may consequently lead to increased cellular cDDP accumulation and enhanced cytotoxicity. In the present study, we found that expression of MRP1 in COC1/DDP cells was suppressed by cotreatment of emodin with cDDP indicating that emodin might synergize with cDDP anticancer effect through downregulation of MRP1. Furthermore, our observation that such MRP1 suppression could be reversed by NAC treatment supports the concept that emodin sensitizes the cDDP resistance cells to cDDP via generation of ROS and ROS-mediated inhibition of MRP1 expression. Precise mechanisms that govern this drug interaction at the molecular level and possible potentiation of other platinum drugs by emodin to overcome MRP1-associated resistance clearly merit future investigation.

Overall, these findings implicate potential application of emodin as a sensitizing agent for cDDP-based therapy and the combinative therapeutic strategy including emodin or other ROS-producing agents may be a beneficial approach to treating refractory ovarian carcinomas resistant to platinum drugs.
